# “Rendezvous technique” for intraluminal vacuum therapy of anastomotic leakage of the jejunum

**DOI:** 10.1186/s40792-016-0243-5

**Published:** 2016-10-18

**Authors:** K. Krajinovic, S. Reimer, T. Kudlich, C. T. Germer, A. Wiegering

**Affiliations:** 1 Department of General, Visceral, Vascular and Pediatric Surgery, University Hospital, Julius-Maximilians-University, University of Wuerzburg, Oberduerrbacherstr. 6, 97080 Wuerzburg, Germany; 2 Department of Internal Medicine II, University of Wuerzburg Medical Center, Oberduerrbacherstr. 2, 97080 Wuerzburg, Germany; 3 Comprehensive Cancer Centre Mainfranken, University Hospital, University of Wuerzburg, Josef-Schneiderstr. 6, 97080 Wuerzburg, Germany; 4 Department of Biochemistry and Molecular Biology, University of Wuerzburg, Oberduerrbacherstr. 2, 97074 Wuerzburg, Germany

**Keywords:** Endosponge, Anastomotic leakage

## Abstract

**Background:**

Anastomotic leakage (AL) is one of the most common and serious complications following visceral surgery. In recent years, endoluminal vacuum therapy has dramatically changed therapeutic options for AL, but its use has been limited to areas easily accessible by endoscope.

**Case presentation:**

We describe the first use of endoluminal vacuum therapy in the small intestine employing a combined surgical and endoscopic “rendezvous technique” in which the surgeon assists the endoscopic placement of an endoluminal vacuum therapy sponge in the jejunum by means of a pullback string. This technique led to a completely closed AL after 27 days and 7 changes of the endosponge.

**Conclusion:**

The combined surgical and endoscopic rendezvous technique can be useful in cases of otherwise difficult endosponge placement.

## Background

Anastomotic leakage (AL) is one of the most common and serious complications following visceral surgery. AL arising early postoperative are amenable to surgical revision, but those occurring later on are difficult to treat. In such cases, surgical revision is often not possible or only possible with major collateral damage due to the presence of intra-abdominal adhesions. In recent years, endoluminal vacuum therapy has been used for late occurring and not surgically amenable AL of the upper gastrointestinal tract (esophagus, stomach). In this procedure, an endosponge is used to treat a perforation in the esophagus or an AL without surgical revision [1, 2]. For technical reasons, however, the use of endoluminal vacuum therapy on perforations and AL has been limited to areas easily accessible by endoscope, which is not the case for AL in the small intestine.

We describe here the first use of endoluminal vacuum therapy in the small intestine employing a combined surgical and endoscopic “rendezvous technique” in which the surgeon assists the endoscopic placement in the jejunum by means of a pullback string.

## Case presentation

**Fig. 1:**
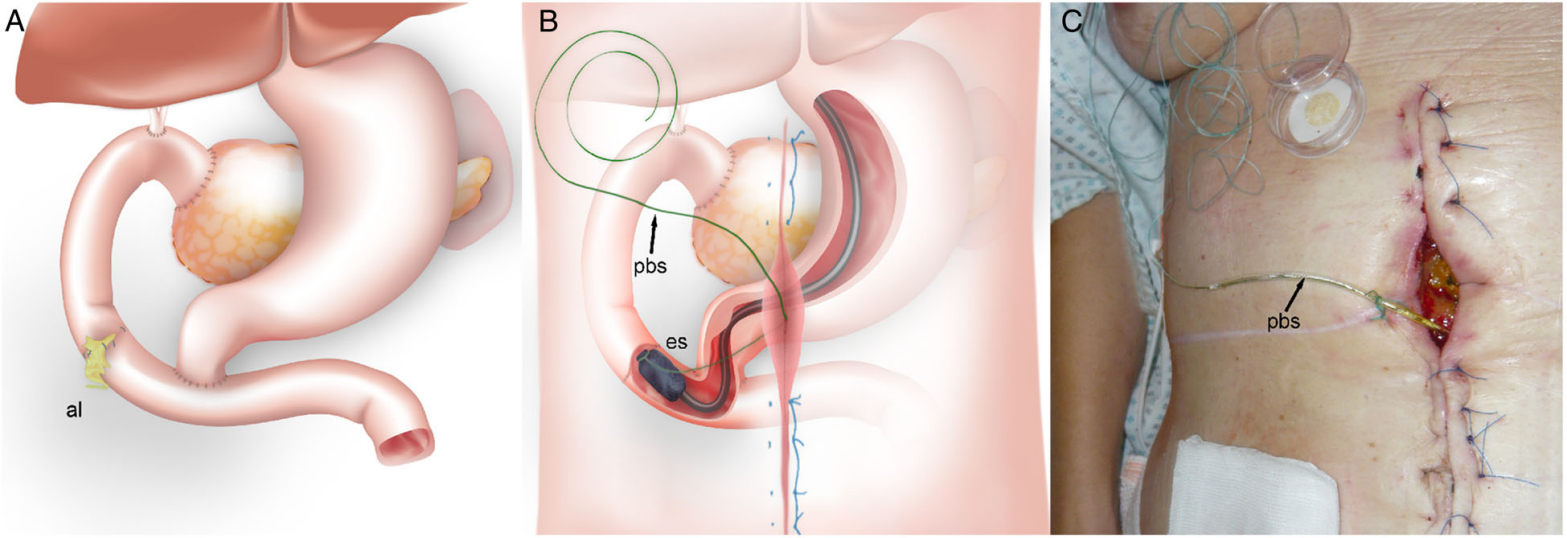
**a** Detailed drawing of the situation after pylorus-preserving pancreatoduodenectomy showing the anastomotic leakage (AL) located in the biliopancreatic loop. **b** Detailed drawing of the modified “rendezvous technique.” *es* endosponge, *pbs* pull back string. **c** View of the external part of the pullback string (*pbs*)

**Fig. 2:**
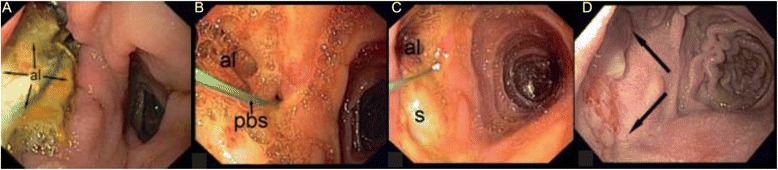
Series of endoscopic images of the anastomotic leakage (AL). **a** Large AL on the ventral side of the jejunal anastomosis. **b** Day 12 of vacuum therapy: the AL is smaller with no necrosis. **c** Day 20 of vacuum therapy: a scar(s) indicating partial recovery of the former leakage. **d** Day 27: end of vacuum therapy with a healed leakage site

A 59-year-old female patient with familial adenomatous polyposis presented with elevated cholestasis parameters. The medical history showed a status post proctocolectomy, and pylorus-preserving pancreatoduodenectomy for duodenal adenoma with high-grade intraepithelial neoplasia. Endoscopy revealed a large sessile adenoma completely occluding the biliopancreatic loop. Despite the total abdominal adhesions, segmental resection was possible during a laparotomy. The anastomosis was performed in hand-sewn end-to-end technique. Due to the total abdominal adhesions and the location, restoring of the intestinal continuity was a technically challenging maneuver. A 6-cm-diameter adenocarcinoma of the jejunum was diagnosed histologically (pT3, G2, R0). Postoperatively, the patient developed a large anastomotic leak on the ventral side of the jejunum on postoperative day 8 with loss of intestinal exudate at the midline incision (Fig. 1a). Surgical treatment was not possible due to the massive intra-abdominal adhesions. The use of intraluminal vacuum therapy was discussed but discounted as too difficult due to the narrowness of the pylorus and the acute angulation of the jejunal loop in which the AL was located. Due to missing therapeutic options in this complicated situation, an individual treatment option was extensively discussed with the patient and she gave her verbal and written consent. It was decided to attempt endoluminal vacuum therapy using a modified rendezvous technique (surgically assisted endoscopy) to place an endosponge at the site of the AL. Briefly, a nonabsorbable “pullback string” was inserted into the jejunal lumen through the visible AL, grasped by the endoscope, pulled back, and the endosponge then attached to the string. Under endoscopic viewing, the endosponge was positioned at the AL site by drawing on the pullback string using a “cableway” guidance system. Negative pressure was applied (100 mmHg). The distal end of the pullback string was left to extend 1.5 m outside the laparotomy wound (Fig. 1b, c). To exchange the endosponge, the old sponge was withdrawn orally, cut off, and the new sponge attached to the pullback string. The endoscopist then pushed the endosponge through the pyloric region while the assistant pulled the distal end of the pullback string to ideally reposition the endosponge in the AL. The endosponge was changed every 4 days. The sessile AL had completely closed after 27 days and 7 changes of the endosponge (Fig. 2). During the treatment period, the patient was admitted to the intermediate care unit (ICU) of our hospital. The nutrition of the patient was carried out by parenteral nutrition and closely monitored (input and output, serum chemistries, triglycerides, and others). “Premixed” standard parenteral nutrition products were used and administered via a central venous port catheter. The patient was allowed to drink clear liquids like tea and water to a limited amount.

### Discussion

Introduction of endoluminal vacuum therapy for AL located in the upper gastrointestinal tract has expanded the available therapeutic options for AL patients and greatly improved their prognosis. To date, however, this technique has been limited to AL that are easily accessible by endoscopy. The modified rendezvous technique described here enables effective treatment even in patients with endoscopically difficult or impossible to reach AL. Often, small intestinal leaks or insufficiencies of the duodenal stump are only detected when they are no longer amenable to surgical treatment. To the best of our knowledge, the literature contains only one report of a similar combined internal/external procedure and a few cases of application in the small intestine [3–5]. In that case, the defect was also successfully treated. Endoluminal vacuum therapy is superior to external drains such as “Robinson” or “Jackson Pratt” drains in this section of the intestine because it can continuously remove the high daily volume of bile, pancreatic fluid, and intestinal fluid passing through, leading to faster healing. It may also lessen the risk of developing an undrained “abscess.” The major limitation of this technique is the need to admit the patient to the ICU ward during the procedure as leaking or technical errors in the system must be detected rapidly to avoid compromising the therapeutic goal. A second disadvantage is that the pullback string by its nature creates a fistula between the abdominal wall and jejunum. But by the time, the leakage site had healed to a small channel of approximately 3 mm in diameter, comparable to the fistula left after removal of a jejunal feeding tube and finally closed spontaneously after completing the endoluminal vacuum therapy.

## Conclusions

In conclusion, our innovative combined intraluminal and extraluminal approach for vacuum therapy using a modified rendezvous technique is an effective way to manage, otherwise difficult to access AL in the small intestine.

## References

[1] Heits N, Stapel L, Reichert B (2014). Endoscopic endoluminal vacuum therapy in esophageal perforation. Ann Thorac Surg.

[2] Seyfried F, Reimer S, Miras AD (2013). Successful treatment of a gastric leak after bariatric surgery using endoluminal vacuum therapy. Endoscopy.

[3] Glatz T, Fischer A, Hoeppner J, Thimme R, Walker C, Richter-Schrag HJ (2015). Vacuum sponge therapy using the pull-through technique via a percutaneous endoscopic gastrostomy to treat iatrogenic duodenal perforation. Endoscopy.

[4] Mennigen R, Senninger N, Laukoetter MG (2014). Novel treatment options for perforations of the upper gastrointestinal tract: endoscopic vacuum therapy and over-the-scope clips. World J Gastroenterol.

[5] Loske G (2014). Intraluminal endoscopic vacuum therapy following ischemia of the blind end of a jejunal Roux-en-Y loop…. Endoscopy.

